# Modulation of flavonoid metabolites in *Arabidopsis thaliana* through overexpression of the *MYB75* transcription factor: role of kaempferol-3,7-dirhamnoside in resistance to the specialist insect herbivore *Pieris brassicae*

**DOI:** 10.1093/jxb/eru096

**Published:** 2014-03-11

**Authors:** Nawaporn Onkokesung, Michael Reichelt, Arjen van Doorn, Robert C. Schuurink, Joop J.A. van Loon, Marcel Dicke

**Affiliations:** ^1^Laboratory of Entomology, Wageningen University, PO Box 8031, 6700EH Wageningen, The Netherlands; ^2^Department of Biochemistry, Max Planck Institute for Chemical Ecology, Hans-Knöll Straβe 8, D-07745 Jena, Germany; ^3^Keygene NV, Agro Business Park 90, 6708OW, Wageningen, The Netherlands; ^4^Plant Physiology, Swammerdam Institute for Life Sciences, University of Amsterdam, Science Park 904, 1098XH Amsterdam, The Netherlands

**Keywords:** Anthocyanins, *Arabidopsis*, flavonols, kaempferol glycosides, *Pieris brassicae*, quercetin glycosides.

## Abstract

Anthocyanins and flavonols are secondary metabolites that can function in plant defence against herbivores. In *Arabidopsis thaliana*, anthocyanin and flavonol biosynthesis are regulated by MYB transcription factors. Overexpression of *MYB75* (oxMYB75) in *Arabidopsis* results in increasing anthocyanin and flavonol levels which enhances plant resistance to generalist caterpillars. However, how these metabolites affect specialist herbivores has remained unknown. Performance of a specialist aphid (*Brevicoryne brassicae*) was unaffected after feeding on oxMYB75 plants, whereas a specialist caterpillar (*Pieris brassicae*) gained significantly higher body mass when feeding on this plant. An increase in anthocyanin and total flavonol glycoside levels correlated negatively with the body mass of caterpillars fed on oxMYB75 plants. However, a significant reduction of kaempferol-3,7-dirhamnoside (KRR) corresponded to an increased susceptibility of oxMYB75 plants to caterpillar feeding. *Pieris brassicae* caterpillars also grew less on an artificial diet containing KRR or on oxMYB75 plants that were exogenously treated with KRR, supporting KRR’s function in direct defence against this specialist caterpillar. The results show that enhancing the activity of the anthocyanin pathway in oxMYB75 plants results in re-channelling of quercetin/kaempferol metabolites which has a negative effect on the accumulation of KRR, a novel defensive metabolite against a specialist caterpillar.

## Summary

The *MYB75* transcription factor is a regulator of anthocyanin biosynthesis. Overexpressing *MYB75* results in re-channelling of quercetin/kaempferol metabolites including reduced accumulation of kaempferol-3,7-dirhamnoside, a novel defensive metabolite against a specialist caterpillar.

## Introduction

Flavonoids are a diverse group of plant secondary metabolites that are widely distributed throughout the plant kingdom (Winkel-Shirley, 2001; Stracke *et al.*, 2007; Martens *et al.*, 2010; Hichri *et al.*, 2011). In terrestrial plants, nine subclasses of flavonoids, namely chalcones, flavones, flavonols, dihydroflavonols, flavandiols, anthocyanins, proanthocyanidins, isoflavonoids, and aurones, have been distinguished based on their chemical structures (Winkel-Shirley, 2001; Martens *et al.*, 2010). The functions of flavonoids in plants range from physiological development (i.e. pigmentation, and seed coat and pollen tube development) to plant responses to abiotic (UV-irradiation and mineral deficiency) and biotic (pathogen infection and herbivory) stresses (Simmonds, 2003; Treutter, 2005; Stracke *et al.*, 2007; Misra *et al.*, 2010; Page *et al.*, 2012). Moreover, due to their health-promoting effects such as antioxidative, anticancer, anti-inflammatory, cardioprotective, and neuroprotective activities in mammalian tissues, flavonoids are also considered to be of pharmaceutical interest (Harborne and Williams, 2000; Winkel-Shirley, 2001; Martens *et al.*, 2010). Because of the diverse functions in plants and mammals, the flavonoid biosynthetic pathway has been extensively studied in various model plants such as snapdragon (*Antirrhinum majus*), petunia (*Petunia hybrida*), and *Arabidopsis thaliana*. Consequently, most of the biosynthetic genes and the transcriptional control of the flavonoid pathway in these model plants have been well documented (Beld *et al.*, 1989; Routaboul *et al.*, 2006; Stracke *et al.*, 2007; Yonekura-Sakakibara *et al.*, 2008; Hichri *et al.*, 2011).

**Fig. 1.**
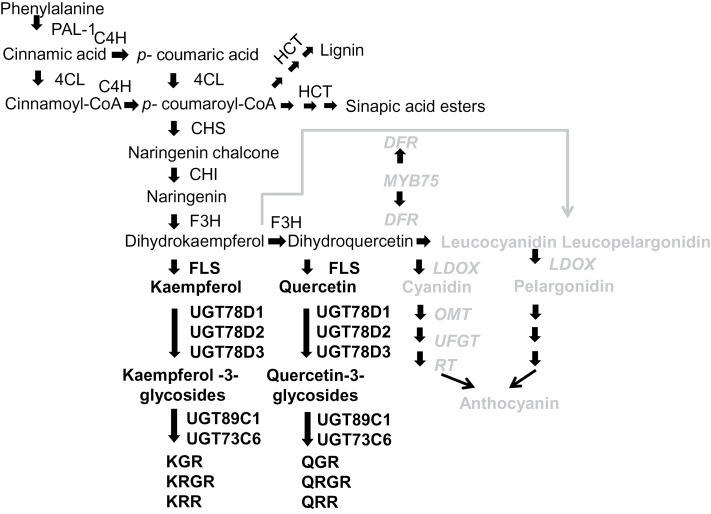
Phenylpropanoid and flavonoid biosynthetic pathways in *Arabidopsis thaliana.* Phenylpropanoid and flavonol biosynthesis is indicated in black. Anthocyanin biosynthesis is indicated in grey. PAL-1, phenylalanine ammonium lyase-1; 4CL, 4-coumarate CoA ligase; C4H, cinnamate-4-hydroxylase; HCT, hydroxycinnamoyl-CoA shikimate/quinate hydroxycinnamoyl transferass; CHS, chalcone synthase; CHI, chalcone isomerase; F3H, flavanone 3-hydroxylase; F3′H, flavonoid 3′-hydroxylase; FLS, flavonol synthase; DFR, dihydroflavonol 4-reductase; LDOX, leucoanthocyanidin dioxygenase; OMT, *O*-methyltransferase; UFGT, UDPG-flavonoid glucosyl transferase; RT, rhamnosyl transferase; UGT, UDP-dependent glycosyltransferase; KGR, kaempferol 3-*O*-glucoside 7-*O*-rhamnoside; KRGR, kaempferol 3-*O*-[6″-*O*-(rhamnosyl) glucoside] 7-*O*-rhamnoside; KRR, kaempferol 3-*O*-rhamnoside 7-*O*-rhamnoside; QGR, quercetin 3-*O*-glucoside 7-*O*-rhamnoside; QRGR, quercetin 3-*O*-[6″-*O*-(rhamnosyl) glucoside] 7-*O*-rhamnoside; QRR, quercetin 3-*O*-rhamnoside 7-*O*-rhamnoside. The figure is based on information from Stracke *et al.* (2007) and Besseau *et al.* (2007).

Flavonoid biosynthesis (Fig. 1) starts with the conversion of *p*-coumaroyl-CoA to naringenin chalcone by chalcone synthase (CHS). Naringenin chalcone is then catalysed by chalcone isomerase (CHI), followed by flavanone 3-hydroxylase (F3H), to form dihydrokaempferol (DHK). DHK is converted to the aglycone kaempferol, a basic flavonol structure, by flavonol synthase (FLS). Alternatively, DHK can also be converted to dihydroquercetin (DHQ) by flavonoid 3′-hydroxylase (F3′H). DHQ is then converted by dihydroflavonol 4-reductase (DFR) to leucocyanidin for anthocyanin biosynthesis. Conversely, DHQ can also be converted by FLS to the aglycone quercetin to enter the flavonol pathway (Winkel-Shirley, 2001; Stracke *et al.*, 2007; Fig. 1). Glycosylation, acylation, and methylation of aglycone structures are important steps in the formation of the diverse and stable structures of anthocyanins and flavonols (Tohge *et al*., 2005, 2007; Yonekura-Sakakibara *et al.*, 2008; Fig. 1).

At the transcriptional level, anthocyanin and flavonol biosynthetic genes are regulated by different transcription factor (TF) families including MYB, basic helix–loop–helix (bHLH), WD-40, and WRKY (Broun, 2005; Ramsay and Glover, 2005; Hichri *et al.*, 2011). Among those families, the MYB family is regarded as a major regulator of the flavonoid pathway (Broun, 2005; Stracke *et al.*, 2007; Dubos *et al.*, 2010). *Production of Anthocyanin Pigment-1* (PAP-1) or *MYB75* is an R2R3 MYB TF gene that was identified by Borevitz *et al.* (2000) as a major regulator of anthocyanin biosynthesis in *Arabidopsis*. Overexpression of *MYB75* by activation tagging resulted in hyperaccumulation of anthocyanins in leaves, roots, stems, and flowers of *Arabidopsis* (Borevitz *et al.*, 2000). Metabolomic and transcriptomic analyses of *MYB75*-overexpressing *Arabidopsis* (oxMYB75) revealed that *MYB75* not only regulated the anthocyanin pathway, but was also involved in the regulation of the flavonol pathway (Tohge *et al*., 2005, 2007; Bhargava *et al.*, 2010). In order to control the anthocyanin biosynthetic pathway, MYB75 forms a complex with bHLH and WD40-repeat protein (MYB/bHLH/WD40), and this complex binds directly to the anthocyanin biosynthetic genes (Ramsay and Glover, 2005; Gonzalez *et al.*, 2008). It has long been suggested that anthocyanin biosynthesis was regulated by jasmonate (JA) signalling. Indeed, a mutant with an altered JA receptor protein, CORONATINE INSENSITIVE-1 (COI-1), exhibited a low accumulation of anthocyanins after exogenous treatment with methyl jasmonate (Qi *et al.*, 2011). Recently, the interaction between the MYB75/bHLH/WD40 complex and Jasmonate ZIM-domain (JAZ) proteins was shown to attenuate anthocyanin biosynthesis (Qi *et al.*, 2011). Moreover, Pourcel *et al.* (2013) recently provided evidence for negative interactions between JA and flavonoid pathways.

JAs are a major group of signalling molecules that regulate plant growth, development, and responses to abiotic and biotic stresses (Pieterse and Dicke, 2007; Wasternack, 2007; Kazan and Manner, 2008; Ballaré, 2011; de Geyter *et al.*, 2012; Erb *et al.*, 2012). Plants perceive the JA signal through the COI-1 receptor. The binding of the JA–isoleucine conjugate (JA-Ile) to the COI-1 receptor leads to the degradation of JAZ repressor proteins that are bound to TFs, and consequently to the activation of JA-responsive genes (Chini *et al.*, 2007; Staswick 2007; Pauwels and Goossens, 2011). The *coi-1* mutant showed a loss in the expression of JA-responsive genes which negatively affected plant growth, fertility, and resistance to pathogens and herbivores (Xie *et al.*, 1998; Devoto *et al.*, 2002; Reymond *et al.*, 2004; Mewis *et al.*, 2005). Attack by herbivores is a major biotic stress for terrestrial plants (Dicke, 2009). In order to survive this stress, plants have evolved various defences including the production of secondary metabolites such as alkaloids, terpenoids, flavonoids, and glucosinolates (Schoonhoven *et al.*, 2005; Hopkins *et al.*, 2009; Dicke *et al.*, 2009; Dicke and Baldwin, 2010; Mithöfer and Boland, 2012).

Glucosinolates (GSs) are typical defensive metabolites in the Brassicaceae family (Mewis *et al.*, 2005; Halkier and Gershenzon, 2006; Winde and Wittstock, 2011). Increased accumulation of GSs in response to herbivory negatively affects the growth and development of generalist herbivores; however, GSs are less effective against specialist herbivores due to the detoxification ability of these herbivores (Wheat *et al.*, 2007; Müller *et al.*, 2010; Winde and Wittstock, 2011; Schramm *et al.*, 2012). Furthermore, GSs can be exploited by specialist insect herbivores to select suitable host plants (de Vos *et al.*, 2008; Hopkins *et al.*, 2009; Sun *et al.*, 2009). Due to the ability of specialist herbivores to tolerate GSs, it is essential that brassicaceous plants use additional strategies to defend themselves against such specialist herbivores. In oxMYB75 *Arabidopsis* and in tobacco plants overexpressing AtMYB75, which accumulate high levels of anthocyanins and flavonols, negative effects have been reported on the survival and the development of the generalist caterpillars *Spodoptera frugiperda*, *S. litura*, and *Helicoverpa armigera* (Johnson and Dowd, 2004; Malone *et al.*, 2009). Flavonols such as rutin and its glycosylated form are reported to enhance the mortality rate and inhibit the growth of *S. litura* caterpillars on groundnut (*Arachis hypogaea* L.) and *Arabidopsis* (Mallikarjuna *et al.*, 2004; Misra *et al.*, 2010). These studies indicate that flavonoids, in addition to GSs, also contribute to plant defence against generalist caterpillars. However, information on the effects of flavonoids on specialist herbivores remains elusive. Moreover, little is known about the defence-related function of flavonoids against herbivores from other feeding guilds, especially phloem feeders, such as aphids. Indeed, it might be advantageous for plants to use the same class of metabolites as defensive compounds against different groups of herbivores.

In this study, experiments were carried out to investigate whether the alteration of anthocyanin and flavonol levels through the overexpression of MYB75 in *Arabidopsis* has an effect on plant defence against a specialist caterpillar (*Pieris brassicae*) and a specialist phloem feeder (the aphid *Brevicoryne brassicae*). Unexpectedly, resistance to caterpillar feeding was significantly lower, while no effect on resistance to aphid feeding was recorded in oxMYB75 plants compared with that in wild type (WT) plants. Flavonol analysis revealed that the kaempferol-3,7-dirhamnoside (KRR) level in oxMYB75 plants was uninduced after caterpillar feeding but significantly decreased after aphid feeding compared with the levels in WT plants. As a significantly lower KRR level after caterpillar feeding correlated with enhanced caterpillar performance on oxMYB75 plants, the role of *MYB75* in regulating KRR accumulation was investigated and KRR was identified as a component of direct defence against specialist caterpillars in *Arabidopsis*.

## Materials and methods

### Plant materials

Two *A. thaliana* genotypes were used: (i) Columbia-0 (Col-0) as the WT; and (ii) overexpression of *production of anthocyanin pigment-1*/*MYB75* in the Col-0 background (oxMYB75) (Borevitz *et al.*, 2000). The oxMYB75 seeds were purchased from the European Arabidopsis Stock Centre (NASC, Nottingham, UK). Seeds were surface-sterilized in a 5ml solution of 10% sodium hypochlorite supplemented with 1% Tween-20 for 5min and subsequently washed three times with sterilized deionized water. Seeds were germinated on half-strength Murashige and Skoog (MS) medium containing 3% sucrose. Plates were kept in the dark at 4 °C for 2 d before transfer to a growth chamber at 21±1 °C; 60±5% relative humidity (RH), 120 μmol m^–2^ s^–1^ light intensity, and an 8:16 (light:dark) photoperiod for 14 d. Seedlings were subsequently transplanted into round plastic pots (diameter 4.5cm) containing sterilized substrate mix (Horticoop, The Netherlands) and kept under environmental conditions as described above.

### Insects

Caterpillars (*P. brassicae* and *Mamestra brassicae*) and aphids (*B. brassicae* and *Myzus persicae*) were obtained from their respective stock colonies of the Laboratory of Entomology, Wageningen University, The Netherlands. Both caterpillars and aphids were reared on Brussels sprout plants (*Brassica oleracea* var. *gemmifera* cv Cyrus) at 22±1 °C, 60±5% RH, 16:8 (light: dark) photoperiod.

### Herbivore performance

Four-week-old WT and oxMYB75 plants were transferred from the short (8:16 light:dark) to long (16:8 light:dark) photoperiod conditions, at 21±1 °C, 60±5% RH, 48h before starting caterpillar or aphid experiments.

A freshly hatched *P. brassicae* or *M. brassicae* neonate caterpillar was placed on a fully expanded leaf of either WT or oxMYB75 plants (one caterpillar per plant). Both specialist and generalist caterpillars were allowed to feed freely on the plants. The weight of individual caterpillars fed on each plant was recorded after 4, 7, 9, and 11 d of feeding. The experiment had 25 replicates per caterpillar species.

Two first-instar aphid nymphs of either *B. brassicae* or *M. persicae* were placed on WT or oxMYB75 plants and the plants were kept in cylindrical plastic containers (diameter 8 cm×height 14cm) covered with fine mesh gauze under long photoperiod conditions at 21±1 °C, 60±5% RH. After 7 d of feeding, the plants were examined and one aphid was removed if both aphids had survived, so that only a single aphid remained. The remaining adult aphid was allowed to feed for another 10 d under the same environmental conditions, and the aphid progeny on each plant was counted. The experiment had 20 replicates per aphid species.

### Plant treatments

For caterpillar treatment, 4-week-old plants were transferred from short to long photoperiod conditions at 21±1 °C, 60±5% RH conditions, 48h prior to the treatment, and plants were kept under these conditions throughout the experiments. First-instar *P. brassicae* larvae were given time to adjust to *Arabidopsis* by feeding on WT plants during the 24h prior to the experiments. Five first-instar larvae of *P. brassicae* were each placed on a fully expanded leaf of one plant. Caterpillar-damaged leaves were collected after 1, 3, 6, 24, and 48h of feeding. Control plants remained undamaged. Caterpillars were removed and leaf tissues for phytohormone and transcript expression analysis were harvested and pooled from two individual plants to obtain one biological replicate. Five biological replicates of caterpillar-damaged or control plants were harvested, flash-frozen in liquid nitrogen, and kept at –80 °C until analysed. For secondary metabolite (glucosinolate, flavonol, and anthocyanin) analysis, five first-instar *P. brassicae* caterpillars were placed on the leaves of 4-week-old WT and oxMYB75 plants. Plants were kept under the same environmental conditions mentioned above for 4 d. Caterpillars were removed and all the aerial tissues were harvested and flash-frozen in liquid nitrogen. Control plants were undamaged plants that had been kept under the same environmental conditions. Tissues were harvested as described above and were kept at –80 °C until analysed.

For aphid treatment, first-instar *B. brassicae* nymphs were given time to adjust to *Arabidopsis* by feeding on WT plants for 24h prior to the experiments. Twenty first-instar *B. brassicae* nymphs were distributed on each plant. Aphid-infested plants were kept in closed cylindrical plastic containers as described above under ‘Herbivore performance’ and kept under the same environmental conditions as for caterpillar treatments. Leaf tissues were collected after 3, 6, 24, and 72h of feeding. Aphids were removed before harvesting tissues. Control plants were untreated plants kept in closed containers under the same environmental conditions. Leaf tissues for phytohormone and transcript analysis were harvested and kept at –80 °C as described for the caterpillar experiments. For secondary metabolite analysis, 20 first-instar *B. brassicae* nymphs were allowed to feed on WT and oxMYB75 plants for 7 d under the same environmental conditions as previously described. Control plants were also kept in similar, closed plastic containers for 7 d under the same environmental conditions. After removing the aphids, all aerial tissues were harvested, flash-frozen in liquid nitrogen, and stored at –80 °C until analysis.

### Phytohormone analysis

Frozen leaf tissues were homogenized by grinding with a mortar and pestle in liquid nitrogen. A 1ml aliquot of ethyl acetate containing internal standards for JA (200ng of D_2_-JA; C/D/N isotopes Inc., Cananda) and salicylic acid (SA) (40ng of D_4_-SA; Sigma-Aldrich, The Netherlands) was added to 0.1g of finely pulverized tissue of each sample and vortexed for 10min. Samples were centrifuged at 16 000 *g* for 30min (4 °C), and the supernatant was transferred to a clean Eppendorf tube. The remaining leaf tissues were re-extracted with 0.5ml of ethyl acetate without internal standards. The supernatant was combined and evaporated by a vacuum concentrator at 30 °C to dryness. The dried samples were resuspended in 0.5ml of 70% methanol (MeOH) and centrifuged at 16 000 *g* for 15min (4 °C). Samples of 0.2ml were transferred to 2ml glass vials for analysis with a liquid chromatography quadrupole tandem mass spectrometry (MS/MS) system (LC/MS; Varian, Palo Alto, CA, USA). A 10 μl aliquot of sample was injected into a Kinetix column (C18, 50×2mm, 2.6 μM, Phenomenex, The Netherlands) connected to a pre-column (C18, 4 mm×2 mm: Phenomenex). The mobile phase consisted of solvent A [0.05% (v/v) formic acid/water] and solvent B (0.05% formic acid/MeOH) used in gradient mode (5% solvent B for 1.5min; a linear gradient to 95% solvent B for 3min, 95% solvent B for 8min; and 5% solvent B for 2.5min). JA, JA-Ile, and SA were detected in the ESI (electrospray ionization) negative mode. Quantification of JA-Ile was performed by integrating peak areas.

### Glucosinolate analysis

Approximately 20mg of lyophilized tissue was used for GS extraction as described in Burow *et al.* (2006). Briefly, 1ml of 80% MeOH containing 0.05mM ρ-hydroxybenzylglucosinolate as internal standard was added to 20mg of tissue and incubated for 10min. Samples were centrifuged at 2500 *g* for 10min, and the supernatants were loaded on columns containing 0.4ml of a 10% (w/v) suspension of DEAE Sephadex A25 in water. Columns were washed with 1ml of 80% (v/v) MeOH, 1ml of water, and 1ml of 0.02M MES buffer (pH 5.2), before 50 μl of sulphatase solution were applied (Hogge *et al.*, 1988). After incubation at room temperature overnight, desulphated GSs were eluted with 1ml of water. A 50 μl aliquot of each sample was analysed by a high-performance liquid chromatography (HPLC; Agilent HP1100 Series) instrument equipped with a C-18 reversed phase column (Nucleodur Sphinx RP, 250×4.6mm, 5 μm particle size, Macherey-Nagel, Germany). The mobile phase consisted of water (solvent A) and acetonitrile (solvent B) used in gradient mode at a flow rate of 1ml min^–1^ at 25 °C. The gradient was as follows: 1.5% B (1min), 1.5–5% B (5min), 5–7% B (2min), 7–21% B (10min), 21–29% B (5min), 29–43% B (7min), 43–100% B (2.5min), 100–1.5% B (0.1min), and 1.5% B (4.9min). The eluent was monitored by diode array detection between 190nm and 360nm (2nm interval). Desulphoglucosinolates were identified based on comparison of retention times and UV absorption spectra with those of known standards. Results are given as μmol g^–1^ dry weight calculated from the peak areas at 229nm relative to the peak area of the internal standard using the relative response factor of 2.0 for aliphatic and 0.5 for indolic GSs.

### Anthocyanin analysis

A 1ml aliquot of 1% HCl (v/v) in 40% MeOH was added to 100mg of lyophilized tissue and thoroughly mixed. The mixture was centrifuged at 16 000 *g* for 15min (4 °C). The upper phase was collected and 1ml of chloroform was added to remove chlorophyll. Samples were centrifuged twice at 16 000 *g* for 15min, and the aqueous phase was collected. Samples were subjected to spectrophotometry and the absorption at 535nm and 650nm was measured. Anthocyanin content was reported as (*A*_535_–*A*_650_) g^–1^ dry matter (DM)

### Flavonol and sinapic acid ester analysis

Samples of 20mg of lyophilized tissue were extracted in 1ml of 80% MeOH. Samples were centrifuged at 2500 *g* for 10min. Then 100 μl aliquots of the supernatants were diluted with 300 μl of water, and 20 μl of each sample was analysed by an HPLC (Agilent HP1100 Series) instrument equipped with a C-18 reversed phase column (Nucleodur Sphinx RP, 250×4.6mm, 5 μm particle size; Macherey-Nagel, Germany). The mobile phase consisted of 0.2% formic acid (v/v) (solvent A) and acetonitrile (solvent B) used in gradient mode at a flow rate of 1ml min^–1^ at 25 °C.The gradient was as follows: 100% A (5min), 0–45% B (15min), 45–100% B (0.1min), 100% B (1.9min), and 100% A (3.9min). The eluent was monitored by a photodiode array detector at 330nm. Kaempferol glycosides were quantified based on an external standard curve of an authentic standard of KRR (High-Purity Compound Standard GmbH, Cunnersdorf, Germany) applying a relative molar response factor of 1.0. Quercetin glycosides were quantified based on an external standard curve of an authentic standard of quercetin-3-glucoside (Sigma-Aldrich, Taufkirchen, Germany) applying a relative molar response factor of 1.0. Sinapoyl malate was quantified based on an external standard curve of an authentic standard sinapic acid (Fluka, Buchs, Switzerland) applying a relative molar response factor of 1.0. The compounds were identified based on UV visible absorption and on mass spectra from LC-MS analysis on a Bruker Esquire 600 IonTrap mass spectrometer (LC conditions were the same as for HPLC-UV analysis) in comparison with the identified metabolites in the literature (Tohge *et al.*, 2005; Yonekura-Sakakibara *et al.*, 2007).

### Quantitative real-time PCR

Total RNA was isolated from ~100mg of finely ground frozen leaf tissue using a NucleoSpin RNA plant kit following the manufacturer’s protocol (Macherey-Nagel, Germany). Total RNA samples were treated with RQ1 DNase (Promega, The Netherlands), followed by ethanol precipitation. cDNA was synthesized from 1 μg of RNA using an iScript cDNA synthesis kit for quantitative real-time PCR (RT-qPCR; Bio-Rad, The Netherlands) in a 20 μl reaction volume. RT-qPCR was performed in Rotor-Gene Q (Qiagen, The Netherlands) in a total volume of 20 μl containing 1.5 μl of cDNA from 1 μg of RNA, 10 μl of iQ SYBR green supermix (Bio-Rad, The Netherlands), and 1.2 μl of 5 μM forward and reverse gene-specific primers. The primer sequences used in this study are listed in Supplementary Table S1 available at *JXB* online. The reactions were run in a three-step program including melting curve; pre-incubation at 95 °C for 10min; amplification for 40 cycles (95 °C for 15 s, 59 °C for 30 s, and 72 °C for 60 s; and melting analysis from 65 °C to 95 °C). For normalization, specific primers of elongation factor-1α from *Arabidopsis* (AtEF-1α; accession NM_001125992) were used. All reactions were performed with five biological replicates. Relative gene expression (2^–ΔΔCt^) was calculated according to Livak and Schmittgen (2001) and Pfaffl (2001).

### Biological activity of kaempferol-3,7-dirhamnoside

To test the biological activity of KRR, an agar-based artificial diet for *Pieris* spp was prepared as described in David and Gardiner (1966) and Junnikkala (1980). Synthetic KRR (High-Purity Compound Standards GmbH) was dissolved in 2% MeOH, and 2ml of 2.5mM KRR solution was added to 50ml of artificial diet to obtain a final concentration of 100 μM KRR in the diets. A control diet was supplied with 2ml of 2% MeOH. A cube (3×3×3cm) of artificial diet was placed in a glass Petri dish and five *P. brassicae* neonates were allowed to feed from it. The diet cube was renewed at 2 d intervals. Average body mass of five caterpillars was measured after 7 d of feeding. The experiments had 10 replicate groups of caterpillars for control and KRR-containing diet.

To test KRR activity *in planta*, 50 μl of 100 μM KRR solution was pipetted over an oxMYB75 leaf [~50mg fresh matter (FM)] and the solution was evenly distributed over a leaf using a paint brush to yield final concentrations of ~1 μmol g^–1^ DM. A control leaf was treated with 50 μl of 2% MeOH. Both sides of two leaves on each plant were treated 10min prior to caterpillar feeding. A second-instar *P. brassicae* larva that had fed on WT plants for 24h was placed within a clip cage on KRR-treated or control leaves. Caterpillars were allowed to feed for 3 d and the body mass of 15 individual caterpillars fed on control or KRR-treated leaves was measured.

### Statistical analysis

The data were analysed by Student’s *t-*test and analysis of variance (ANOVA) followed by Tukey HSD post-hoc test using SPSS 19 (IBM, Chicago, IL, USA).

## Results

### *MYB75* expression is up-regulated by *P. brassicae* but not by *M. persicae* infestation

**Fig. 2.**
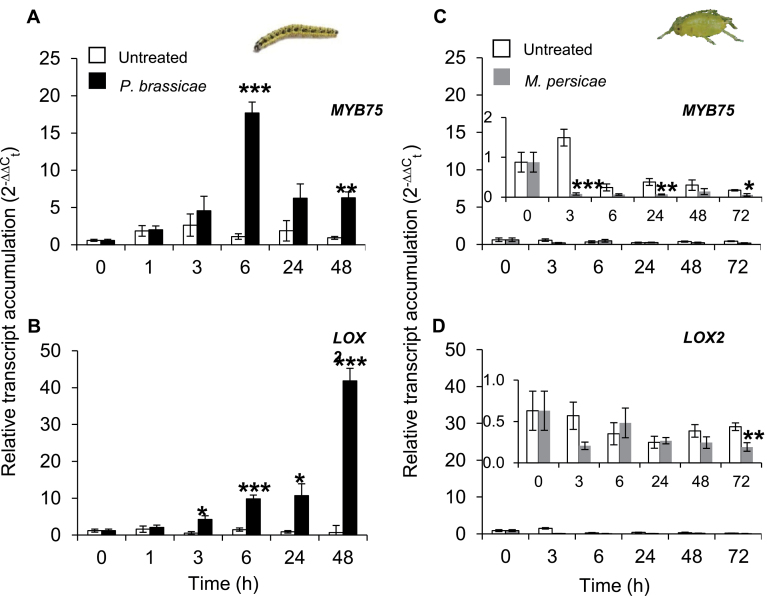
*Pieris brassicae* or *Myzus persicae* feeding differentially affect *MYB75* and *Lipoxygenase-2* (*LOX-2*) transcript expression in *Arabidopsis* Col-0 plants. Relative transcript abundance of *MYB75* and *LOX-2* after specialist caterpillar *P. brassicae* feeding (A, B) and generalist aphid *M. persicae* feeding (C, D) at different time points since inoculation of plants with the herbivores. All data points represent the mean ±SE of four biological replicates. Transcript expression levels of *MYB75* and *LOX-2* were compared between undamaged (control) and herbivory treatment at each time point by Student’s *t-*test; asterisks indicate significant differences; *P≤ 0.05; **P ≤0.01; ***P ≤0.001. (This figure is available in colour at *JXB* online.)

To examine *MYB75* responses to caterpillar or aphid feeding, the time course of the transcript accumulation of *MYB75* after *P. brassicae* or *M. persicae* feeding on *Arabidopsis* Col-0 (WT) plants were analysed by RT-qPCR. Transcript levels of *MYB75* increased transiently after 6h of caterpillar feeding and *MYB75* transcripts remained at significantly higher levels compared with the level in undamaged plants at the latest time point; namely 48h (Fig. 2A). In contrast, aphid feeding significantly suppressed *MYB75* expression compared with control plants as early as at 3h of feeding, an effect which persisted until 72h of aphid feeding (Fig. 2C, inset). The results show that caterpillar and aphid feeding have opposite effects on *MYB75* expression.

JA is an early signalling compound involved in the induction of plant responses to herbivory (Dicke *et al.*, 1999; Halitschke and Baldwin 2003; Dombrecht *et al.*, 2007; Wu and Baldwin, 2010). The expression patterns of *MYB75* and *Lipoxygenase-2* (*LOX-2*), a gene encoding an enzyme that mediates a rate-limiting step in JA biosynthesis, were compared Relative transcript accumulation of *LOX-2* significantly increased in response to caterpillar feeding, whereas *LOX-2* expression in untreated control plants remained essentially unchanged (Fig. 2B). Unlike *MYB75*, *LOX-2* transcripts continued to increase through 48h of feeding. Although aphid feeding suppressed both *LOX-2* and *MYB75* expression, the effect was stronger for *MYB75*; the effect for *LOX-2* was only statistically significant at 72h since the start of aphid feeding (Fig. 2C, D and insets). These results show that the different effects of feeding by caterpillars and aphids on *MYB75* expression extend to *LOX-2* transcripts in *Arabidopsis*. Because the expression patterns of many defence-related genes are correlated with that of *LOX-2* (Halitschke and Baldwin, 2003; Reymond *et al.*, 2004; Bidart-Bouzat and Kliebenstein, 2011), it was hypothesized that *MYB75* plays a role in plant defence against caterpillar and aphid attack.

### Overexpression of *MYB75* in *Arabidopsis* has differential effects on plant resistance to caterpillars and aphids

The potential function of *MYB75* in resistance against caterpillars and aphids was addressed by assessing the performance of specialist and generalist lepidopteran (*P. brassicae* and *M. brassicae*) caterpillars and homopteran (*B. brassicae* and *M. persicae*) aphids on WT and oxMYB75 plants. It was observed that oxMYB75 plants exhibited deep purple leaves only under long-day conditions (16h of light, Supplementary Fig. S1A, B at *JXB* online). Moreover, the relative transcript level of *MYB75* and the anthocyanin accumulation was ~2-fold and ~55-fold higher, respectively, in oxMYB75 than in WT plants kept in long-day conditions. (Supplementary Fig. S1C, D). Therefore, all experiments in this study were done after plants had been transferred to long-day conditions to obtain phenotype uniformity.

**Fig. 3.**
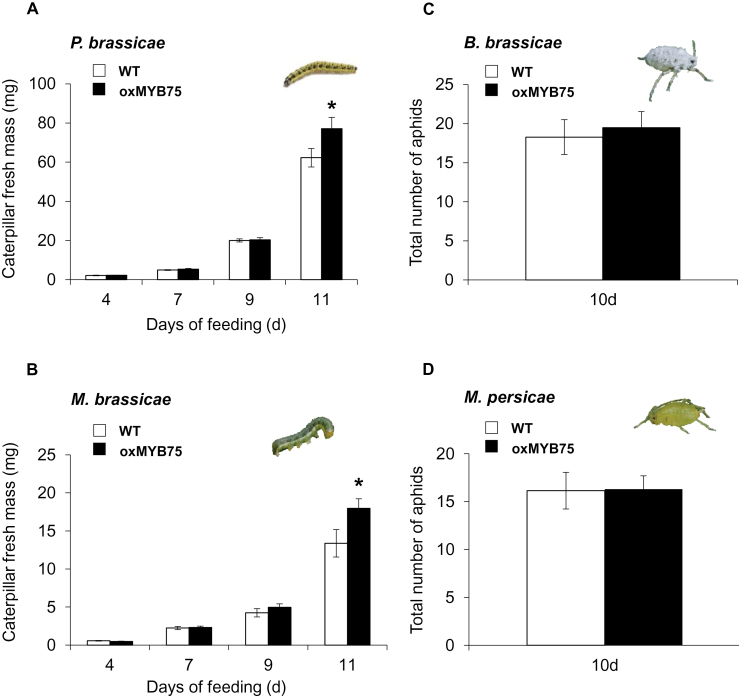
Performance of caterpillars and aphids on WT and oxMYB75 plants. Caterpillar performance is determined by measuring the body mass of 25 individual caterpillars feeding on WT or oxMYB75 plants for 4, 7, 9, and 11 d. The number of aphid progeny from one adult aphid is used as an indicator for aphid performance. (A) Average body mass of *Pieris brassicae* and of (B) *Mamestra brassicae* (mean ±SE, *n*=25) at designated time points feeding on WT or oxMYB75 plants. (C) The number of aphid progeny from one adult aphid of *Brevicoryne brassicae* or (D) of *Myzus persicae* after 10 d of feeding on WT or oxMYB75 plants. The caterpillar mass and aphid progeny number are compared between WT and oxMYB75 plants by Student’s *t*-test; asterisks indicate significant differences *P≤0.05. (This figure is available in colour at *JXB* online.)

Caterpillar and aphid performance on WT and oxMYB75 plants was quantified by measuring fresh body mass as a proxy for caterpillar performance and total offspring production by an adult aphid as an indicator for aphid performance. *Pieris brassicae* and *M. brassicae* caterpillars showed no significant difference in body mass after 4, 7, and 9 d of feeding on either WT or oxMYB75 plants (Fig. 3A, B). However, both caterpillar species gained significantly more body mass after 11 d of feeding on oxMYB75 compared with WT plants (Fig. 3A, B; *P. brassicae*, Student’s *t*-test, *P*≤0.05; *M. brassicae*, Student’s *t*-test, *P*≤0.05). The population sizes of *B. brassicae* and *M. persicae* on WT and oxMYB75 plants were similar after 10 d of feeding (Fig. 3C, D). The results on herbivore performance indicate that oxMYB75 plants are more susceptible to caterpillars, but equally resistant to aphid feeding compared with WT plants.

To investigate the effects of *MYB75* on the defence mechanisms against caterpillar and aphid feeding, the levels of typical signalling molecules (phytohormones) and defence metabolites (glucosinolates) were quantified in WT and oxMYB75 plants. Because a similar performance pattern was observed in this study for the two caterpillar species and the two aphid species, despite the difference in the degree of host–plant specialization, further studies were restricted to the specialist species *P. brassicae* and *B. brassicae*.

### Phytohormone and glucosinolate profiles in WT and oxMYB75 plants in response to caterpillar or aphid feeding

JA, JA-Ile, and SA are the major phytohormones that regulate plant responses to caterpillar and aphid attack (Mewis *et al.*, 2005; Thompson and Goggin, 2006; Wu and Baldwin, 2010). To examine the effect of overexpressing *MYB75* on the level of these hormones after herbivore attack, the time courses of JA, JA-Ile, and SA in WT and oxMYB75 plants were analysed by LC/MS. Due to the difference in experimental conditions between *P. brassicae* and *B. brassicae* feeding (see the Materials and methods), two separate experiments were performed, each with a new set of untreated (control) plants.

**Fig. 4.**
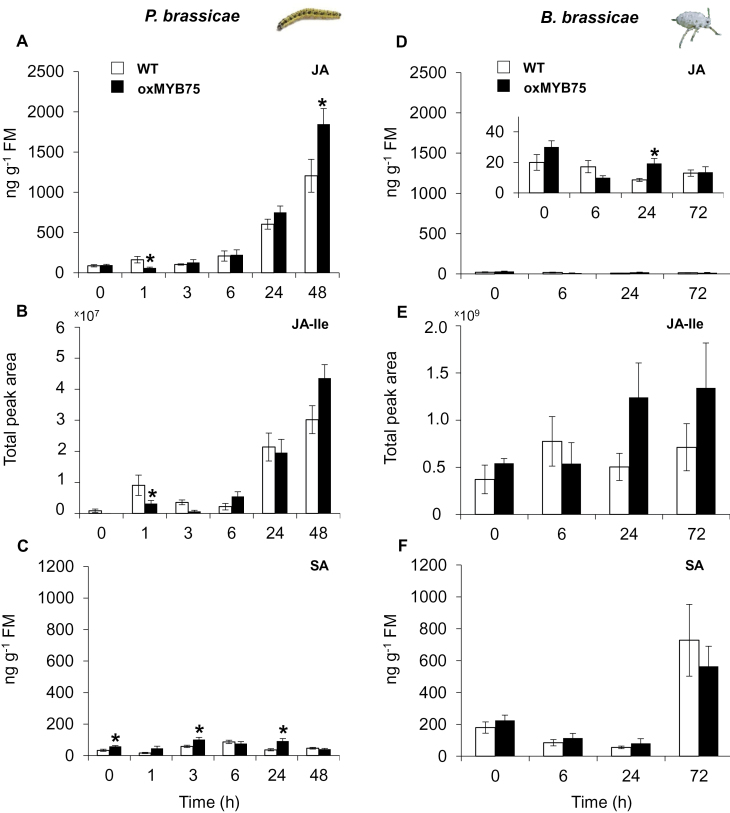
Caterpillar and aphid feeding have opposite effects on phytohormone levels in WT and oxMYB75 plants. (A–C) Mean ±SE (*n*=5) of JA, JA-Ile, and SA levels in WT and oxMYB75 plants at the designated time points after feeding by five first-instar larvae of *P. brassicae*. (E–F) Mean ±SE (*n*=5) of JA, JA-Ile, and SA levels in WT and oxMYB75 plants at different time points in response to feeding by 20 nymphs of *B. brassicae*. Levels of JA, JA-Ile, and SA in the WT and oxMYB75 were compared at the correlated time points by Student’s *t*-test; asterisks indicate a significant difference *P≤0.05. FM, fresh mass. (This figure is available in colour at *JXB* online.)

JA and JA-Ile levels greatly increased relative to basal levels as a response to caterpillar feeding in WT and oxMYB75 plants (Fig. 4A, B). Even though the level of JA in oxMYB75 plants was significantly higher after 48h of feeding compared with WT plants (Student’s *t*-test, *P*≤0.05), the JA-Ile level did not show a significant difference at this time point (Fig. 4A, B). In contrast, SA levels remained relatively low in WT and oxMYB75 plants at all time points of caterpillar feeding, which suggests that *P. brassicae* feeding did not induce SA accumulation in *Arabidopsis* (Fig. 4C). In contrast to the response to caterpillar feeding, JA and JA-Ile levels changed slightly in WT and oxMYB75 plants after aphid feeding (Fig. 4D, E). However, SA levels were strongly enhanced after 72h of aphid feeding in both plant types (Fig. 4F).

**Fig. 5.**
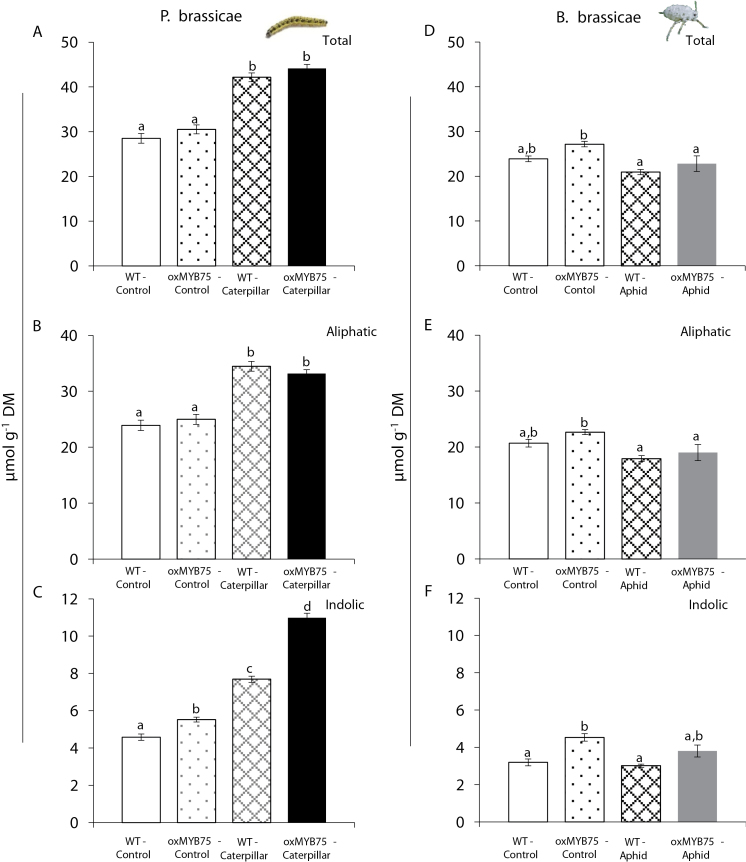
Glucosinolate levels in WT and oxMYB75 plants in response to caterpillars and aphids. Mean ±SE (*n*=5) levels of total, aliphatic, and indolic glucosinolates in WT and oxMYB75 plants (A–C) after 4 d of feeding by *P. brassicae* caterpillars and (D–F) after 7 d of feeding by the aphid *B. brassicae*. Glucosinolate levels in control and herbivory treatment from WT and oxMYB75 plants were compared by ANOVA; mean values having no letters in common differ significantly (Tukey HSD post-hoc test; *P*≤0.05). DM, dry mass. (This figure is available in colour at *JXB* online.)

Increasing GS levels in *Arabidopsis* after herbivore feeding requires a signal from the JA pathway (Mikkelsen *et al.*, 2003; Mewis *et al.*, 2005). The slightly higher JA level in oxMYB75 plants after caterpillar and aphid feeding prompted the investigation of whether there was an effect of herbivory on GS accumulation in the overexpressing plants. Caterpillar feeding significantly increased total GS levels in WT and oxMYB75 plants from the basal levels (ANOVA, *P*≤0.001; Fig. 5A–C). Interestingly, while the aliphatic GS level was comparable between WT and oxMYB75 plants, oxMYB75 plants contained significantly higher total indolic GS levels compared with WT plants (ANOVA, *P*≤0.001) at the constitutive and caterpillar-induced levels (Fig. 5B, C). In addition, the significantly higher expression levels of two genes involved in indolic GS biosynthesis, *CYP79B2* and *CYP79B3*, in oxMYB75 correlated with the higher indolic GS level in the transgenic plants (Supplementary Fig. S4A, B at *JXB* online). In contrast, there is no significant difference in GS levels before and after aphid feeding in WT plants, while the levels of total, aliphatic, and indolic GSs in oxMYB75 plants were significantly decreased in response to aphid feeding and the levels in aphid-infested oxMYB75 plants were similar to the levels in WT plants (Fig. 5D–F). It has been reported that 4-methoxyindol-3-ylmethylglucosinolate (4MOI3M), an indolic GS, was an effective deterrent against the generalist aphid (*M. persicae*) in *Arabidopsis* (Kim and Jander, 2007; Pfalz *et al.*, 2009). It is interesting that 4MOI3M levels were not significantly different between WT and oxMYB75 plants before and after aphid feeding (Supplementary Fig. S4B) which corresponds to the similar aphid performance observed on WT and oxMYB75 plants (Fig. 3C, D).

Although some significant differences in phytohormone and GS levels were recorded between oxMYB75 and WT plants after caterpillar or aphid feeding, these differences do not seem to explain why oxMYB75 plants are more susceptible to caterpillars but have similar resistance to aphids compared with WT plants (Fig. 3). Because *MYB75* is a major regulator of the anthocyanin and flavonol pathways, it was hypothesized that changes in anthocyanin and/or flavonol accumulations caused by overexpression of *MYB7*5 modify the plant–herbivore interactions. To test this hypothesis, the transcriptional response of the selected anthocyanin and flavonol biosynthetic genes in WT and oxMYB75 plants were analysed after caterpillar or aphid feeding.

### Caterpillar or aphid feeding differentially affect the expression of anthocyanin and flavonol biosynthetic genes in oxMYB75 plants

To gain information on the effect of overexpression of *MYB75* on anthocyanin and flavonol biosynthetic pathways, the expression of genes encoding enzymes in the anthocyanin (*DFR*) and flavonol (*FLS-1* and *F3′H*) biosynthetic pathways before and after caterpillar or aphid feeding were analysed by RT-qPCR. In addition, expression of *CHS*, a gene encoding an enzyme that synthesizes the core structure of anthocyanins and flavonols, was also examined.

**Fig. 6.**
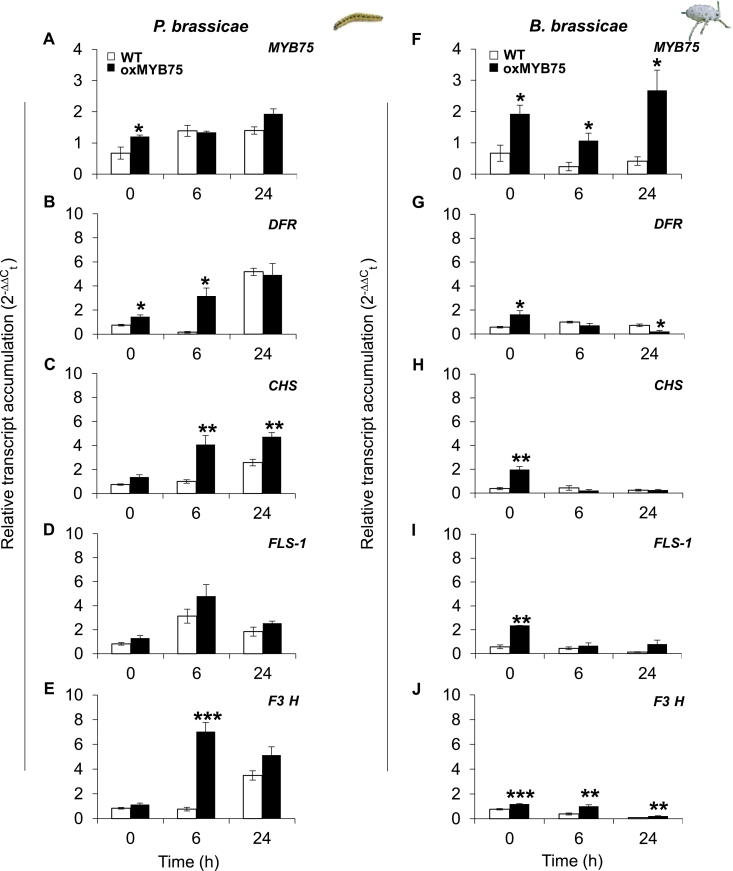
*MYB75* is involved in the regulation of anthocyanin and flavonol biosynthetic genes in *Arabidopsis*. Relative transcript abundance of *MYB75*, *CHS*, *DFR*, *FLS-1*, and *F3′H* in leaf tissues of WT and oxMYB75 after feeding by *P. brassicae* caterpillars (A–E) or *B. brassicae* aphids (F–J) at different time points. All bar values represent the mean ±SE of five biological replicates. Transcript expression levels were compared between the WT and oxMYB75 at the corresponding time points by Student’s *t*-test; asterisks indicate significant differences, *P≤0.05; ** *P*≤0.01; ***P≤0.001. (This figure is available in colour at *JXB* online.)

*MYB75*, *DFR*, *CHS*, *FLS-1*, and *F3′H* transcript levels in WT and oxMYB75 plants were significantly increased after 24h of caterpillar feeding compared with the levels in their respective control plants (Fig. 6A–E). Interestingly, the expression of anthocyanin and flavonol biosynthetic genes showed a significant difference from the uninduced levels in oxMYB75 plants after 6h of feeding (Student’s *t*-test, *P*≤0.05), while significant expression of these genes in WT plants was observed only at 24h of feeding (Student’s *t*-test, *P*≤0.01). In contrast, after 24h of aphid feeding, a suppression of *DFR*, *CHS*, *FLS-1*, and *F3′H* was exhibited whereas *MYB75* transcript expression remained at a high level in oxMYB75 plants (Fig. 6F–J). These results suggest that although *MYB75* can regulate anthocyanin and flavonol biosynthetic genes during caterpillar feeding, aphid feeding seems to suppress the expression of these genes via a *MYB75*-independent mechanism.

### Overexpression of *MYB75* impairs the accumulation of kaempferol glycosides after caterpillar or aphid feeding

**Fig. 7.**
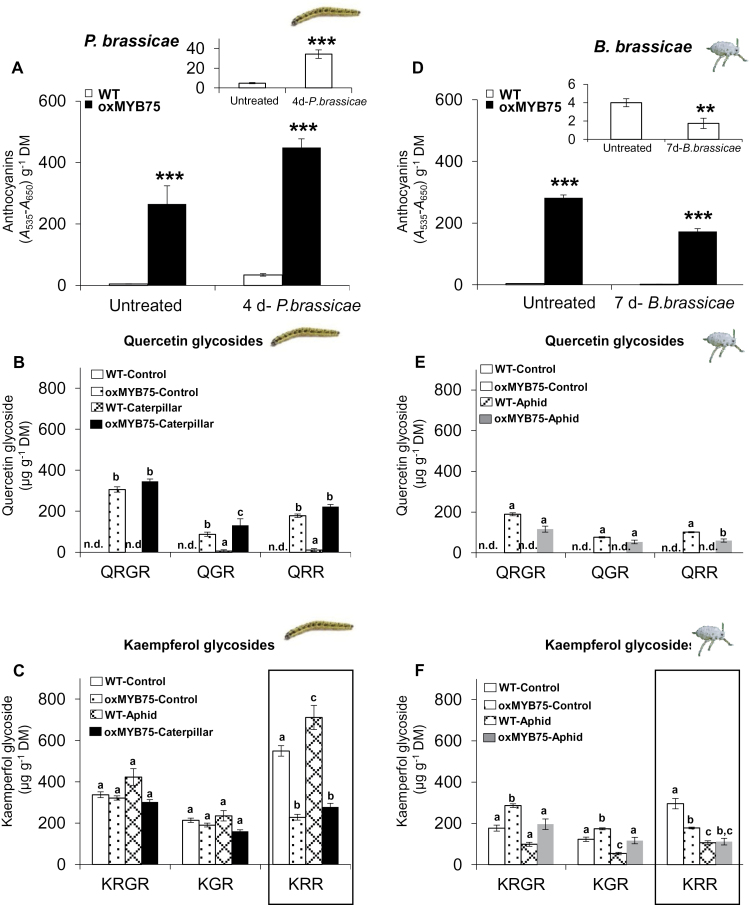
Anthocyanin and flavonol levels are increased after caterpillar feeding but are reduced after aphid feeding in WT and oxMYB75 plants. Anthocyanin levels (mean ±SE, *n*=5) in WT and oxMYB75 plants after feeding by *P. brassicae* caterpillars for 4 d (A) or after feeding by *B. brassicae* aphids for 7 d (D). Levels of quercetin and kaempferol glycoside (mean ±SE, *n*=5) in WT and oxMYB75 plants after *P. brassicae* feeding for 4 d (B, C) or after *B. brassicae* feeding for 7 d (E, F). Values of quercetin and kaempferol glycosides (B, C, E, F) were compared between control and herbivory treatment and between WT and oxMYB75 plants by ANOVA; mean values having no letters in common differ significantly (Tukey HSD post-hoc test; *P*≤0.05). Abbreviations; n.d., not detected; DM, dry mass; QRGR, quercetin 3-*O*-[6″-*O*-(rhamnosyl) glucoside] 7-*O*-rhamnoside; QGR, quercetin 3-*O*-glucoside 7-*O*-rhamnoside; QRR, quercetin 3-*O*-rhamnoside 7-*O*-rhamnoside; KRGR kaempferol 3-*O*-[6″-*O*-(rhamnosyl) glucoside] 7-*O*-rhamnoside; KGR kaempferol 3-*O*-glucoside 7-*O*-rhamnoside; KRR, 3-*O*-rhamnoside 7-*O*-rhamnoside. (This figure is available in colour at *JXB* online.)

To connect the expression of genes involved in the biosynthesis of anthocyanins and flavonols with herbivore performance on oxMYB75 plants, anthocyanin and flavonol levels were quantified in WT and oxMYB75 plants after caterpillar or aphid feeding. The anthocyanin level was significantly increased in WT (Student’s *t*-test, *P*≤0.001) and in oxMYB75 (Student’s *t*-test, *P*≤0.05) plants after 4 d of caterpillar feeding (Fig. 7A and inset). However, the caterpillar-induced anthocyanin level in WT plants was significantly lower (Student’s *t*-test, *P*≤0.001) than in oxMYB75 plants (Fig. 7A). Aphid feeding, in contrast, significantly reduced anthocyanin levels in WT (Student’s *t*-test, *P*≤0.01) and in oxMYB75 (Student’s *t*-test, *P*≤0.001) plants compared with the untreated plants (Fig. 7D and inset).

Next the accumulation of common flavonol metabolites (i.e. quercetin and kaempferol glycosides) in *Arabidopsis* leaves was analysed by HPLC-UV and HPLC/MS (Veit and Pauli, 1999; Tohge *et al.*, 2005) in WT and oxMYB75 plants after caterpillar or aphid feeding. The MS analysis revealed that the levels of three quercetin glycosides, quercetin 3-*O*-[6″-*O*-(rhamnosyl) glucoside] 7-*O*-rhamnoside (QRGR; *m/z* 755, [M-H^–^]^–^), quercetin 3-*O*-glucoside 7-*O*-rhamnoside (QGR; *m/z* 609, [M-H^–^]^–^), and quercetin 3-*O*-rhamnoside 7-*O*-rhamnoside (QRR; *m/z* 593 [M-H^–^]^–^), were not significantly changed after caterpillar or aphid feeding on oxMYB75 plants (Fig. 7B, E). The levels of these quercetin glycosides in WT plants were under the limit of detection before and after herbivore feeding (Fig. 7B, E). Moreover, caterpillar and aphid feeding also rarely affected the levels of two kaempferol glycosides, kaempferol 3-*O*-[6″-*O*-(rhamnosyl) glucoside] 7-*O*-rhamnoside (KRGR; *m/z* 739, [M-H^–^]^–^) and kaempferol 3-*O*-glucoside 7-*O*-rhamnoside (KGR; *m/z* 593, [M-H^–^]^–^), in WT and oxMYB75 plants (Fig. 7C, F). Interestingly, the kaempferol 3-*O*-rhamnoside 7-*O*-rhamnoside (KRR; *m/z* 577 [M-H^–^]^–^) level in WT plants was significantly enhanced (ANOVA, *P*≤0.05) after caterpillar feeding and reduced (ANOVA, *P*≤0.001) after aphid feeding (Fig. 7C, F). However, the KRR level in undamaged oxMYB75 plants was significantly lower than the level in the WT and was unaffected by caterpillar feeding (Fig. 7C). Furthermore, aphid feeding reduced the KRR level in oxMYB75 to the same level as in aphid-infested WT plants (Fig. 7F). In addition, because a redirection of the metabolic flux between the flavonol and sinapate ester branch of the phenylpropanoid pathway has been reported in *Arabidopsis* (Peer *et al.*, 2001; Besseau *et al.*, 2007; Demkura and Ballaré, 2012; Fig.1), the level of sinapoyl malate, an abundant sinapate ester in *Arabidopsis* leaves, was quantified. There was no significant difference in sinapoyl malate levels between WT and oxMYB75 plants, either before or after caterpillar or aphid feeding (Supplementary Fig. S5 at *JXB* online). The results indicate that overexpressing *MYB75* influences the redirection of metabolic flux between anthocyanins and flavonols but does not affect the flux between flavonols and sinapoyl esters.

In conclusion, similar trends are observed for the production of anthocyanins, flavonols, and sinapoyl malate in WT and oxMYB75 plants (Fig. 7; Supplementary Fig. S5 at *JXB* online). Only for a specific kaempferol glycoside, namely KRR, was a significant difference recorded between WT and oxMYB75 plants before and after caterpillar feeding (Fig. 7C). Importantly, the difference in KRR level between WT and oxMYB75 plants after caterpillar feeding corresponds to the caterpillar performance on the respective plant types (Fig. 3A, B). Therefore, it was explicitly investigated whether KRR is a direct defence metabolite that negatively affects *P. brassicae* caterpillars.

### Effect of KRR on *P. brassicae* caterpillars

**Fig. 8.**
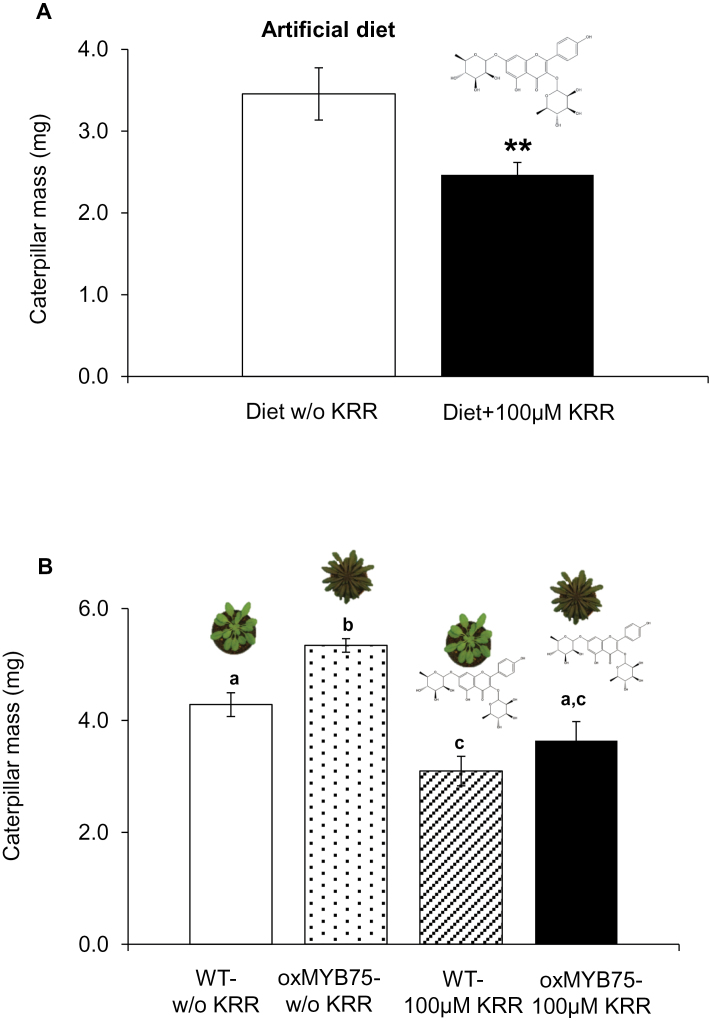
KRR incorporated in an artificial diet and exogenously applied on intact leaves of oxMYB75 and WT plants negatively affects *P. brassicae* caterpillar growth. (A) Mean ±SE (*n*=10) of *P. brassicae* caterpillar body mass after 7 d of feeding on artificial diet containing 0 or 100 μM KRR. The caterpillar mass is compared between control and KRR-containing diet by Student’s *t*-test; asterisks indicate significant differences, **P≤0.01. (B) Mean ±SE (*n*=15) of second-instar *P. brassicae* caterpillar body mass fed on two intact leaves of oxMYB75 or the WT treated with 1 μmol g^–1^ DM mM KRR in 2% MeOH for 3 d. The caterpillar mass was compared between control and KRR treatment and between WT and oxMYB75 plants by ANOVA; mean values having no letters in common differ significantly (Tukey HSD post-hoc test; *P*≤0.05). w/o KRR, without KRR. (This figure is available in colour at *JXB* online.)

To investigate a defence-related function of KRR, body mass of *P. brassicae* caterpillars feeding since egg hatch for 7 d on artificial diet containing KRR was assessed. As the highest KRR level in WT plants after *P. brassicae* caterpillar feeding was ~711 μg g^–1^ DM (~1.2 μmol g^–1^ DM; KRR mol. wt 578.52), 100 μM KRR was supplied to the artificial diets to obtain a similar KRR concentration as reached after caterpillar feeding in WT plants. Interestingly, caterpillars had significantly lower body mass when fed on the diets containing KRR (Fig. 8A) compared with those fed on the diet without KRR (Student’s *t*-test, *P*≤0.01). As there may be an interaction between KRR and other metabolites within oxMYB75 leaves, KRR activity was also tested *in planta* by exogenous application of KRR onto oxMYB75 leaves. The combination of concentration and volume that was exogenously applied resulted in a final concentration of 1 μmol KRR g^–1^ DM on oxMYB75 leaves prior to caterpillar feeding. Again, caterpillar body mass was significantly lower when they fed on leaves treated with 1 μmol KRR g^–1^ DM (ANOVA, *P*≤0.01) compared with the group fed on the control oxMYB75 leaves (Fig. 8B). It is interesting that the body mass of caterpillars fed on KRR-treated oxMYB75 leaves was comparable with that of caterpillars fed on untreated WT leaves (Fig. 8B). Moreover, a significant reduction of caterpillar body mass was also observed when caterpillars fed on KRR-treated WT leaves (ANOVA, *P*≤0.05) when compared with the group fed on untreated WT leaves (Fig. 8B). Together, these results further support KRR’s function in the direct defence against specialist caterpillars.

## Discussion

Although the role of anthocyanins and flavonols in plant responses to abiotic stresses has been well described (Lea *et al.*, 2007; Quina *et al.*, 2009; Page *et al.*, 2012), little is known of their role in plant–herbivore interactions (Simmonds, 2003; Schoonhoven *et al.*, 2005). Inhibition of growth and development reported for several generalist caterpillar species feeding on *Arabidopsis* and tobacco overexpressing *MYB75* suggests that anthocyanins and flavonols play a role in plant defence against herbivores (Johnson and Dowd, 2004; Malone *et al.*, 2009). Here, it is shown that a reduction of KRR level mediated by overexpression of *MYB75* plays a role in the defence against the specialist caterpillar *P. brassicae* in *Arabidopsis*.

### A regulatory role for *MYB75* in the flavonoid pathway during caterpillar or aphid feeding

Although *MYB75* was originally described as a master regulator for anthocyanin biosynthesis (Borevitz *et al.*, 2000; Gonzalez *et al.*, 2008), microarray studies of oxMYB75 plants revealed the up-regulation of not only anthocyanin biosynthetic genes such as *DFR* and *leucoanthocyanidin dioxygenase* (*LDOX*), but also flavonol biosynthetic genes including *FLS-1*, *F3H*, and *F3′H* in leaf and root tissues (Tohge *et al.*, 2005). Furthermore, the expression of ‘early’ genes in the flavonoid pathway, including *CHS* and *Phenylalanine Ammonia Lyase-1* (*PAL-1*), was also partially controlled by *MYB75* (Tohge *et al.*, 2005; Stracke *et al.*, 2007). The present data show that the expression of *DFR*, *CHS*, *FLS-1*, and *F3′H* was higher in undamaged oxMYB75 than in undamaged WT plants (Fig. 6A–J); however, a significant difference for these transcripts was only found in the aphid experiments (Fig. 6F–J). This phenomenon might occur due to the differences in experimental settings (see the Materials and methods) between the caterpillar and aphid experiments. Nevertheless, the same expression trends (higher in oxMYB75 plants) of these selected biosynthetic genes was recorded for both caterpillar and aphid experiments.

An unexpected reduced expression of genes involved in the anthocyanin and flavonol biosynthetic pathways in oxMYB75 plants after aphid feeding suggests that other regulators might be involved in regulating these pathways during aphid feeding (Fig. 6F–J). In fact, *MYB90* (*PAP-2*) and *MYB32* are reported to co-regulate the anthocyanin pathway with *MYB75* (Borevitz *et al.*, 2000; Ramsay and Glover, 2005; Stracke *et al.*, 2007), and *MYB11*, *MYB12*, and *MYB111* are co-regulators of the flavonol pathway (Stracke *et al.*, 2010). Therefore, a suppression of anthocyanin and flavonol biosynthetic genes after aphid feeding on oxMYB75 plants might be due to the ability of aphids to suppress the effect of *MYB75* overexpression by modulating other MYB regulators of the anthocyanin and flavonol pathways. Analysis of *MYB32*, *MYB90*, *MYB11*, *MYB12*, and *MYB111* expression in oxMYB75 plants before and after aphid feeding is needed to falsify this hypothesis and would provide new insight into how aphids manipulate these metabolic pathways at the transcriptional level in their host plant.

### Overexpression of *MYB75* affects JA levels after caterpillar feeding

JAs are major signalling molecules involved in plant responses to herbivore feeding (Dicke *et al.*, 1999; Halitschke and Baldwin, 2003; Dombrecht *et al.*, 2007; Wu and Baldwin, 2010). In WT and oxMYB75 plants, caterpillar feeding resulted in an increase in JA and JA-Ile levels compared with the basal levels. It is interesting that JA and JA-Ile levels in oxMYB75 plants were significantly lower after 1h of caterpillar feeding compared with the levels in caterpillar-damaged WT plants (Fig. 4A, B). Recently, Pourcel *et al.* (2013) reported the possible temporal negative feedback effect of flavonoids on wound-induced JA and JA-Ile accumulation in a chalcone isomerase (*tt-5*) *Arabidopsis* mutant that fails to accumulate anthocyanins and flavonols. Therefore, the delayed induction of JA and JA-Ile at 1h of feeding might be due to significantly higher basal levels of anthocyanin and flavonol in oxMYB75 plants (Fig.7). In addition, JA and JA-Ile levels in oxMYB75 were also lower at the early time point (6h) of aphid feeding than in WT plants (Fig. 4D, E).

### KRR: a novel defence metabolite against the specialist caterpillar *P. brassicae*

It was observed that oxMYB75 plants were more susceptible to specialist and generalist caterpillars (*P. brassicae* and *M. brassicae*) than WT plants (Fig. 3A, B). Previously, Johnson and Dowd (2004) reported an increased resistance to the generalist caterpillar *S. frugiperda* in oxMYB75 plants which is opposite to the present results (Fig. 3A, B). Differences in growth conditions, use of detached leaves, caterpillar feeding time, and the caterpillar species in the previous study might underlie these differences. The increase in the JA and JA-Ile levels and significantly higher indolic GS levels in oxMYB75 plants would be expected to enhance rather than reduce caterpillar resistance because JAs and GSs are involved in plant resistance against caterpillars (Dicke *et al.*, 1999; Halitschke and Baldwin, 2003; Hopkins *et al.*, 2009). Gigolashvili *et al.* (2007) showed that an increase in the indolic GS level had a negative effect on a generalist caterpillar (*S. exigua*); however, no conclusive evidence of the negative effects of indolic GSs on specialist caterpillars has been reported. Instead, Müller *et al.* (2010) showed that a decreasing indolic GS level in *Arabidopsis* had no effect on performance of the specialist caterpillar *P. rapae*. Taken together, a reduced resistance to specialist *P. brassicae* caterpillars in oxMYB75 plants would result from the change in the mixture of defence metabolites rather than only the change in indolic GS level.

Besides the alteration in phytohormones and indolic GSs after caterpillar or aphid feeding, significant changes in anthocyanin, and quercetin and kaempferol glycoside levels were also observed in oxMYB75 plants (Fig. 7). Quercetin glycoside levels were significantly higher in oxMYB75 than in WT plants; however, the levels of quercetin glycosides were not affected by caterpillar or aphid feeding in either oxMYB75 or WT plants (Fig. 7B, E). Moreover, the caterpillars perform better on oxMYB75 plants than on WT plants (Fig. 3) which argues against a role for the quercetin glycosides in plant defence. In contrast, KRR was the only kaempferol glycoside whose levels were affected by caterpillar and aphid feeding in WT plants (Fig. 7C, F). Furthermore, the KRR level was significantly reduced (49%) in undamaged oxMYB75 plants compared with the level in undamaged WT plants. Herbivore feeding barely affected the KRR level in oxMYB75 plants, while the KRR level was significantly enhanced (30%) in caterpillar-damaged WT plants (Fig. 7C). In fact, the reduction of KRR in high anthocyanin-containing plants has previously been reported in *Arabidopsis* (Gou *et al.*, 2011).

Although there is no available information on the toxicity of KRR to animals, the present study shows that KRR negatively affects the specialist caterpillar *P. brassicae*. A significant reduction in caterpillar body mass was observed when the caterpillars fed on KRR-treated oxMYB75 plants or on artificial medium containing KRR (Fig. 8). In addition, exogenously applied KRR on WT leaves further reduced the body mass of *P. brassicae* caterpillars when compared with the group fed on non-KRR-treated WT leaves (Fig. 8B). Together, the results support a role for KRR in direct plant defence against the specialist caterpillar *P. brassicae*. It is noteworthy that overexpression of *MYB75* in *Arabidopsis* has a pleiotropic effect on the level of flavonol glycosides (Fig. 7). Therefore, further studies using a mutant which abolishes KRR biosynthesis, such as a mutant in *UGT89C1*, a gene encoding 7-*O*-rhamnosyltransferase (Yonekura-Sakakibara *et al.*, 2007), will further confirm KRR’s role in plant–herbivore interactions

It is interesting that although the KRR level was significantly reduced in WT plants to a level comparable with what was recorded in oxMYB75 plants after aphid feeding, aphid population growth did not differ between the two genotypes (Fig. 3C, D). This suggests that an increased accumulation of KRR in foliar tissue does not affect aphid performance. Histochemical detection of KRR in phloem and incorporation of KRR in an artificial diet would provide more information about the possible role of KRR in plant–aphid interactions.

### Conclusion

Plants respond to herbivore attack through a complicated network of cross-talking phytohormone signal transduction pathways, resulting in an equally complex response in terms of transcriptional and metabolic reprogramming (Kessler and Baldwin, 2002; Thompson and Goggin, 2006; Pieterse and Dicke, 2007; Wu and Baldwin, 2010). Even though a role for anthocyanins and flavonols in plant responses to abiotic stresses has been described, little information on their role in plant defences against specialist herbivores is available. Here, overexpression of the *MYB75* TF gene in *Arabidopsis*, which resulted in the excessive production of anthocyanins and flavonols, was used to study the possible role of anthocyanins and flavonols in plant–herbivore interactions. Herbivore performance and chemical analysis of oxMYB75 plants compared with WT plants, in addition to targeted pharmacological experiments, provides evidence that the flavonol glycoside KRR plays a part in mediating resistance against the specialist caterpillar *P. brassicae*. Although a significant reduction in KRR after aphid feeding suggests a function for KRR in plant–aphid interactions, more studies on a KRR-deficient mutant are required to elucidate further KRR functions in interactions between *Arabidopsis* and specialist insect herbivores.

## Supplementary data

Supplementary data are available at *JXB* online.

Figure S1. oxMYB75 phenotypes in short-day and long-day conditions

Figure S2. Aliphatic GSs in WT and oxMYB75 plants as a response to caterpillar or aphid feeding

Figure S3. Indolic GSs in WT and oxMYB75 plants as a response to caterpillar or aphid feeding

Figure S4. Relative transcript expression of selected indolic GS biosynthetic genes.

Figure S5. Sinapoyl malate accumulation in WT and oxMYB75 plants before and after caterpillar or aphid feeding.

Table S1. Specific primer sequences for RT-qPCR.

Supplementary Data
