# Supplementary material for: Modulation of flavonoid metabolites in Arabidopsis thaliana through overexpression of the MYB75 transcription factor: role of kaempferol-3,7-dirhamnoside in resistance to the specialist insect herbivore Pieris brassicae
Source: J Exp Bot. 2014 Mar 11;65(8):2203–17. doi: 10.1093/jxb/eru096 (PMC3991749; doi:10.1093/jxb/eru096)
Supplement: Supplementary Data [file supp_65_8_2203__index.html]

Modulation of flavonoid metabolites in Arabidopsis thaliana through overexpression of the MYB75 transcription factor: role of kaempferol-3,7-dirhamnoside in resistance to the specialist insect herbivore Pieris brassicae — Modulation of flavonoid metabolites in Arabidopsis thaliana through overexpression of the MYB75 transcription factor: role of kaempferol-3,7-dirhamnoside in resistance to the specialist insect herbivore Pieris brassicae — Supplementary Data 

# Modulation of flavonoid metabolites in *Arabidopsis thaliana* through overexpression of the *MYB75* transcription factor: role of kaempferol-3,7-dirhamnoside in resistance to the specialist insect herbivore *Pieris brassicae*

## Supplementary Data

Data files

**Files in this Data Supplement:**

- Supplementary Data - Supplementary Data
