# Supplementary material for: Modulation of flavonoid metabolites in Arabidopsis thaliana through overexpression of the MYB75 transcription factor: role of kaempferol-3,7-dirhamnoside in resistance to the specialist insect herbivore Pieris brassicae
Source: J Exp Bot. 2014 Mar 11;65(8):2203–17. doi: 10.1093/jxb/eru096 (PMC3991749; doi:10.1093/jxb/eru096)
Supplement: Supplementary Data [file supp_eru096_jexbot107557_file001.pdf]

Modulation of flavonoid metabolites in *Arabidopsis thaliana* through overexpression of the *MYB75* transcription factor affects plant responses to specialist tissue-chewing and phloem-feeding herbivores

Nawaporn Onkokesung[1], Michael Reichelt[2], Arjen van Doorn[3], Robert C. Schuurink[4], Joop J.A. van Loon[1] & Marcel Dicke[1*]

[1]Laboratory of Entomology, Wageningen University, PO Box 8031, 6700EH Wageningen, The Netherlands

[2]Department of Biochemistry, Max Planck Institute for Chemical Ecology, Hans-Knöll Straβe 8, D-07745 Jena, Germany

[3]Keygene NV, Agro Business Park 90, 6708OW, Wageningen, The Netherlands

[4]Plant Physiology, Swammerdam Institute for Life Sciences, University of Amsterdam, Science Park 904, 1098XH Amsterdam, The Netherlands

Supplementary data

Supplementary Figure legends

Figure S1. Overexpression of *MYB75* in Arabidopsis increases anthocyanin accumulation. (A) Overexpression of *MYB75* (oxMYB75) results in purple veins against a green background of the leaves when grown under short-day (8 h of light) conditions. (B) deep-purple leaves as reported in Borevitz *et al.* (2000) are observed when oxMYB75 plants grow under long-day (16 h of light) conditions. (C) Relative transcript abundance of *MYB75* (mean ± SE, n = 5) in undamaged WT and oxMYB75 plants grown under long day conditions. (D). Anthocyanin accumulation (mean ± SE, n = 5) in aerial tissues of undamaged WT and oxMYB75 plants grown under long-day conditions. The expression level of *MYB75* and anthocyanin accumulation were compared between WT and oxMYB75 plants by Student's *t*-test; asterisks indicate significant differences* $p \leq 0.05$; ** $p \leq 0.001$.

Figure S2. Aliphatic glucosinolate levels in aerial tissues of WT and oxMYB75 plants in control and feeding damage by *P. brassicae* caterpillars or by *B. brassicae* aphids. Levels of individual aliphatic glucosinolate (mean ± SE, n = 5) in WT and oxMYB75 plants after 4 d feeding of *P. brassicae* caterpillar (A) or after 7 d feeding of *B. brassicae* aphid (B). Values of glucosinolates were compared between WT and oxMYB75 plants and between control and herbivory treatment by ANOVA; mean values having no letters in common differ significantly (Tukey HSD *post hoc* test; $p \leq 0.05$). Abbreviations: 3MSOP, 3-methylsulfinylpropylglucosinolate; 4MSOB, 4-methylsulfinylbutylglucosinolate; 5MSOP, 5-methylsulfinylpentylglucosinolate; 7MSOH, 7-methylsulfinylheptylglucosinolate; 4MTB, 4-methylthiobutylglucosinolate; 8MSOO, 8-methylsulfinyloctylglucosinolate; DM, dry mass.

Figure S3. **Indolic glucosinolate levels are increased as a response to caterpillar feeding but reduced after aphid feeding in WT and oxMYB75 plant.** Levels of individual indolic glucosinolates (mean ± SE, n = 5) in WT or oxMYB75 plants after 4 d of feeding damage by *P. brassicae* caterpillars (A) or after 7d of feeding

damaged by *B. brassicae* aphids (B). Levels of indolic glucosinolates were compared between WT and oxMYB75 plants and between control and herbivory treatment by ANOVA, mean values having no letters in common differ significantly (Tukey HSD *post-hoc* test; $p \leq 0.05$). Abbreviations: I3M, indoyl-3-methylglucosinolate; 4OHI3M, 4-hydroxy indoyl-3-methylglucosinolate; 4MOI3M, 4-methoxy indoyl-3-methylglucosinolate; 1MOI3M, 1-methoxy indoyl-3-methylglucosinolate; DM, dry mass.

Figure S4. Relative expression of selected indolic glucosinolate biosynthetic genes and β-thioglucoside glucohydrolases in WT and oxMYB75 plants after feeding by *P. brassicae* caterpillars or *B. brassicae* aphids. Relative transcript abundance (mean ± SE, n = 5) of indolic glucosinolate biosynthetic genes (*CYP79B2* and *CYP79B3*) and β-thioglucoside glucohydrolases 1 and 2 (*TGG1* and *TGG2*) in WT and oxMYB75 plants after caterpillar feeding (A-D) or after aphid feeding (E-H) at different time points. The transcription levels were compared between WT and oxMYB75 plants at the correlated time point by Student's *t*-test, asterisks indicate significant difference; * $p \leq 0.05$, ** $p \leq 0.01$.

Figure S5. Sinapoyl malate accumulation in WT and oxMYB75 plants in response to *P. brassicae* or *B. brassicae* feeding. Sinapoyl malate concentration (mean± SE, n = 5) in WT and oxMYB75 plants after 4 d of *P.brassicae* caterpillar (A) or after 7 d feeding of *B.brassicae* aphid (B). Sinapoyl malate concentration was calculated based on an external standard curve determined for sinapic acid.

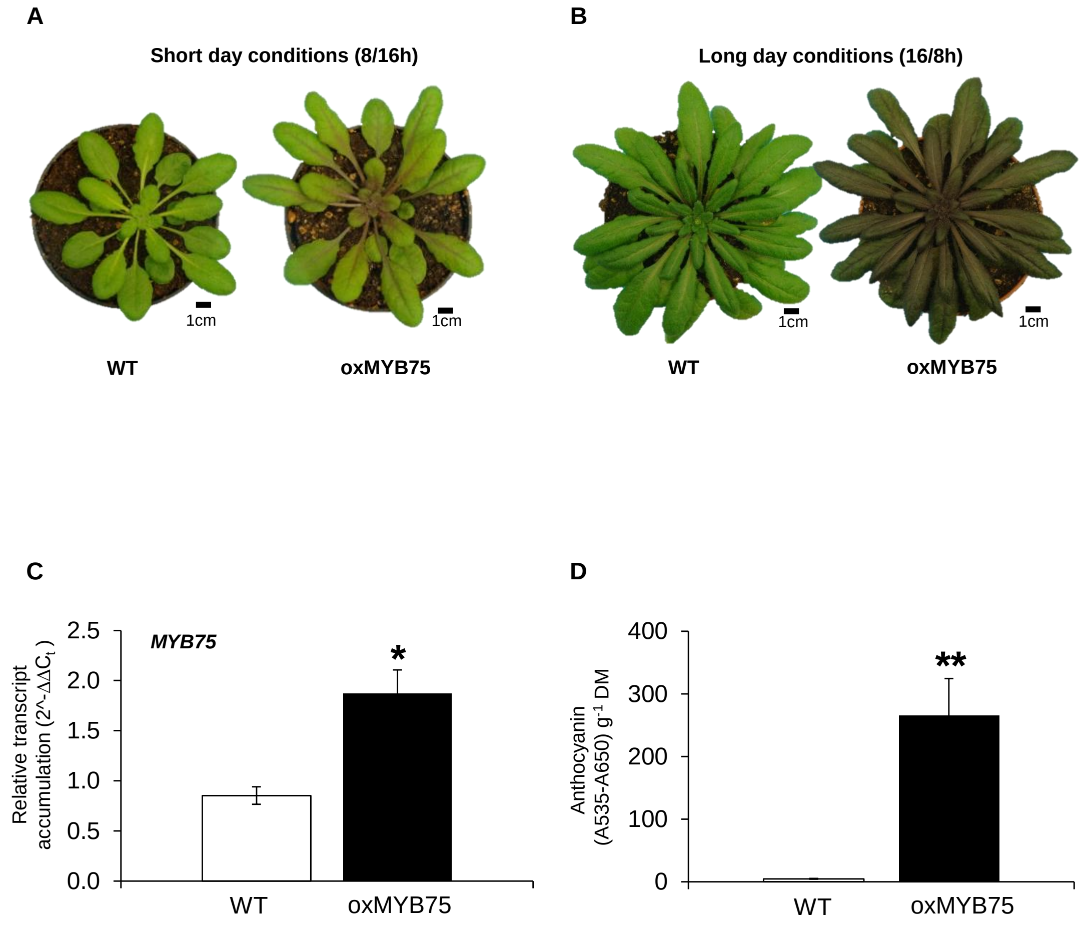

A
Short day conditions (8/16h)
1cm
1cm
WT
oxMYB75
B
Long day conditions (16/8h)
1cm
1cm
WT
oxMYB75
C
MYB75
Relative transcript accumulation (2^-ΔΔCt)
2.5
2.0
1.5
1.0
0.5
0.0
*
WT
oxMYB75
D
Anthocyanin (A535-A650) g-1 DM
400
300
200
100
0
**
WT
oxMYB75

Figure S1.

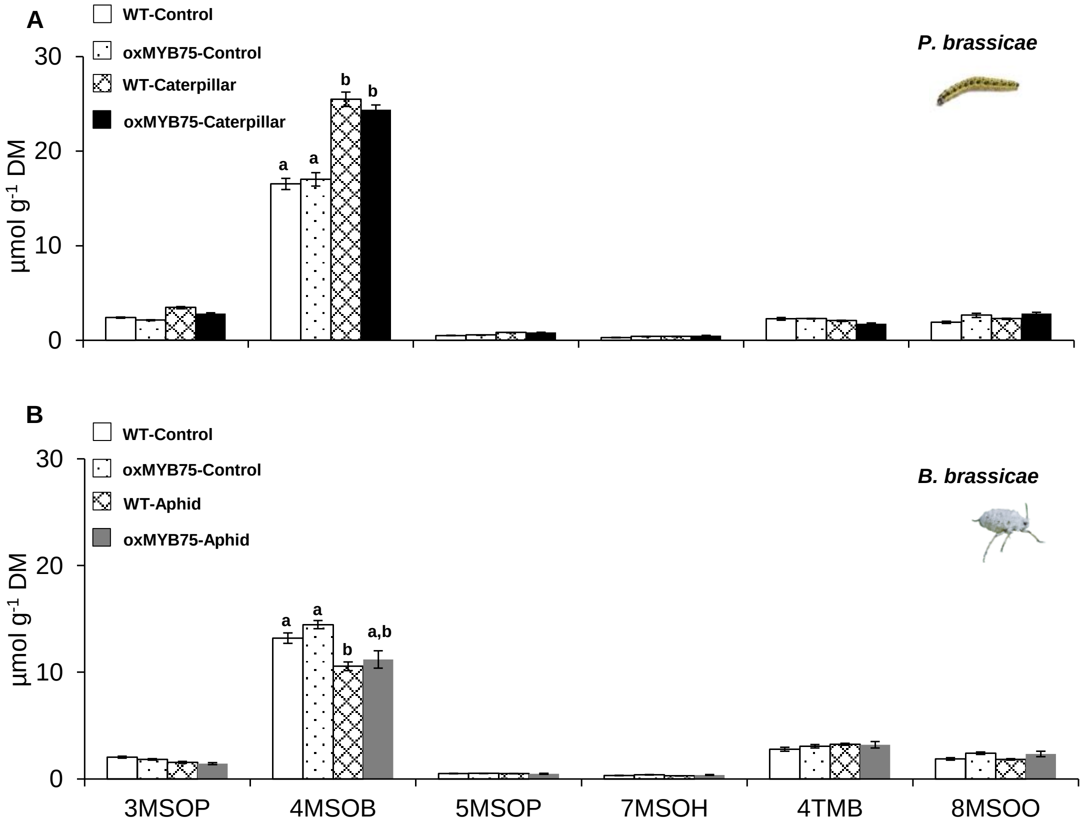

A
WT-Control
oxMYB75-Control
WT-Caterpillar
oxMYB75-Caterpillar
P. brassicae
μmol g-1 DM
30
20
10
0
a
a
b
b
B
WT-Control
oxMYB75-Control
WT-Aphid
oxMYB75-Aphid
B. brassicae
μmol g-1 DM
30
20
10
0
a
a
b
a,b
3MSOP
4MSOB
5MSOP
7MSOH
4TMB
8MSOO

Figure S2.

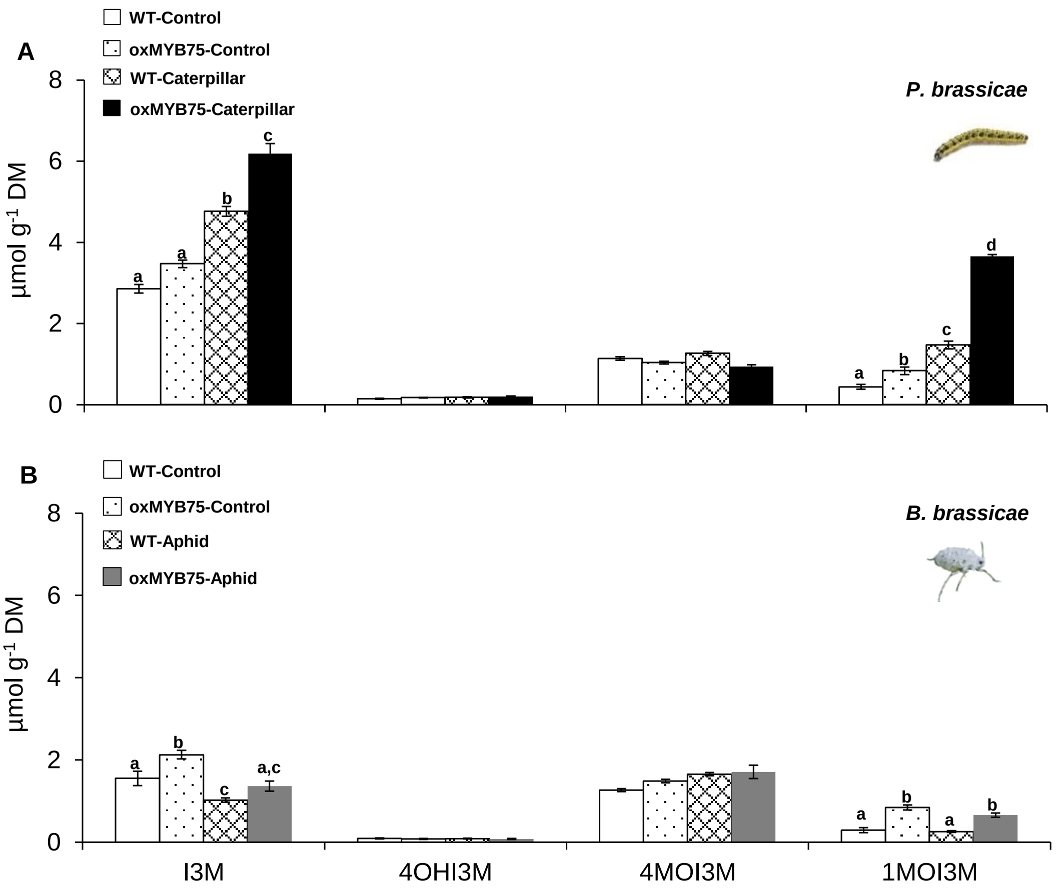
A
WT-Control
oxMYB75-Control
WT-Caterpillar
oxMYB75-Caterpillar
P. brassicae
µmol g-1 DM
8
6
4
2
0
a
a
b
c
a
b
c
d
B
WT-Control
oxMYB75-Control
WT-Aphid
oxMYB75-Aphid
B. brassicae
µmol g-1 DM
8
6
4
2
0
a
b
c
a,c
a
b
a
b
I3M
4OHI3M
4MOI3M
1MOI3M

Figure S3.

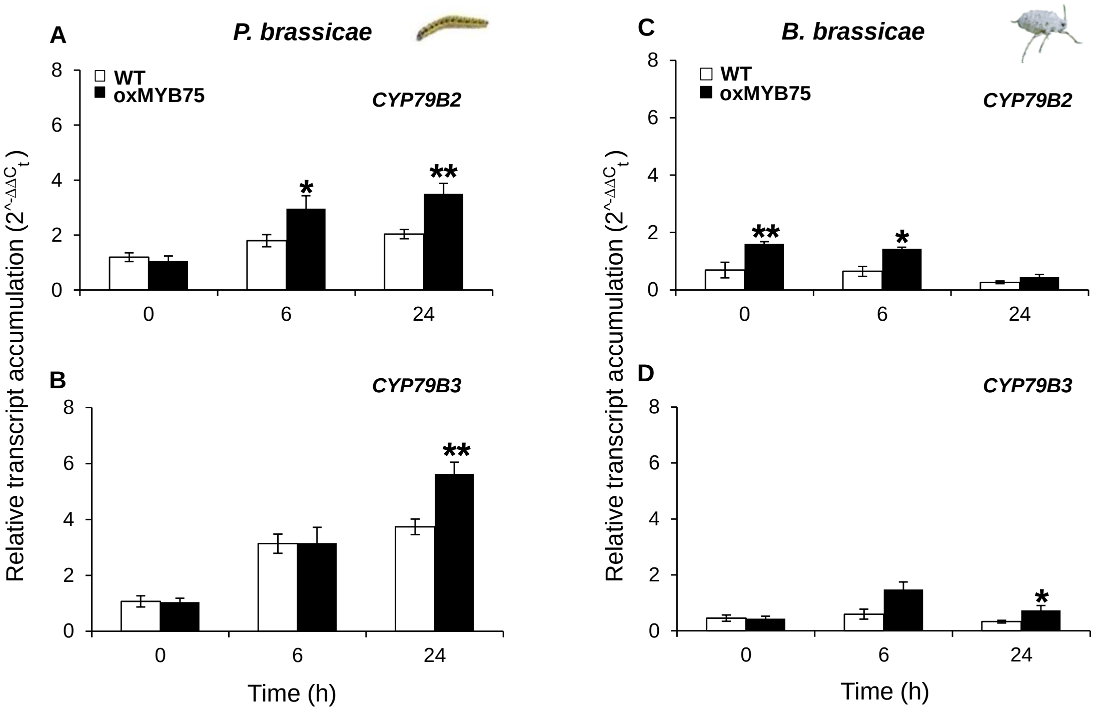

A
P. brassicae
WT
oxMYB75
CYP79B2
B
CYP79B3
C
B. brassicae
D
Relative transcript accumulation (2^-ΔΔCt)
Time (h)
0
6
24
8
4
2
*
**

Figure S4.

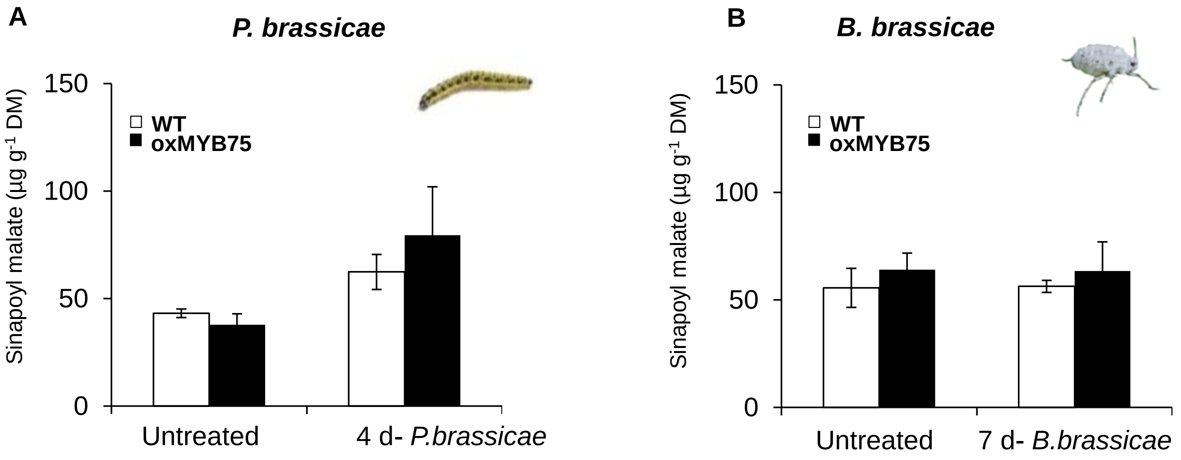
A
P. brassicae
Sinapoyl malate (µg g-1 DM)
150
100
50
0
WT
oxMYB75
Untreated
4 d- P.brassicae
B
B. brassicae
Sinapoyl malate (µg g-1 DM)
150
100
50
0
WT
oxMYB75
Untreated
7 d- B.brassicae

Figure S5.

Table S1. Specific primers used for quantitative real-time PCR. Primer sequences indicated as 5′ → 3′.

Gene	Sequence (5´→3´)
**MYB75-Forward**	GCGAAAAGGTGCTTGGACT
**MYB75-Reverse**	CTAGAAGCCTATGAAGGCGAA
**LOX2-Forward**	ACTTGCTCGTCCGGTAATTGG
**LOX2-Reverse**	GTACGGCCTTGCCTGTGAATG
**DFR-Forward**	GATTTCGCCGAAGAGAAAGGA
**DFR-Reverse**	TGGCGGCTGCTTGTTCG
**FLS-Forward**	ACGGTGGATAAAGAGAAGACGA
**FLS-Reverse**	CACTTCCTATTCCACAACCACA
**CHS-Forward**	CTGACACATCTGTCGGAGAG
**CHS-Reverse**	CAGAAGAGGGAGTTCCAGTC
**F3´H-Forward**	CTCTCGCCGGAGTATTCAAC
**F3´H-Reverse**	CTCCGTCACCGTCAAGATC
